# Protocol for Extraction and Electron Microscopy Visualization of Lipids in *Viburnum tinus* Fruit Using Cryo-Ultramicrotomy

**DOI:** 10.1016/j.xpro.2020.100201

**Published:** 2020-12-09

**Authors:** Miranda Sinnott-Armstrong, Silvia Vignolini, Yu Ogawa

**Affiliations:** 1 Department of Ecology and Evolutionary Biology, Yale University, New Haven, CT 06511, USA; 2 Chemistry Department, University of Cambridge, Lensfield Road, Cambridge CB2 1EW, UK; 3 Univ. Grenoble Alpes, CNRS, Cermav, 38000 Grenoble, France

**Keywords:** Biophysics, Microscopy, Structural Biology

## Abstract

We recently reported that *Viburnum tinus* fruit generates its metallic blue color using globular lipid inclusions embedded in its epicarpal cell walls. This protocol describes steps to visualize the lipidic nature of the nanostructure using cryo-ultramicrotomy, chloroform extraction, and transmission electron microscopy (TEM) imaging. This method is useful to localize and characterize novel lipidic nanostructures embedded in both plant and animal tissues at the TEM resolution.

For complete details on the use and execution of this protocol, please refer to Middleton et al. (2020).

## Highlights

• A protocol for visualizing lipid nanostructures in *Viburnum tinus* fruits is described
• Cryo-microtomy and chloroform extraction allow localizing lipids at nanometric scale
• The protocol is applicable to plants, yeasts, and fungi

## Graphical Abstract

**Figure:**
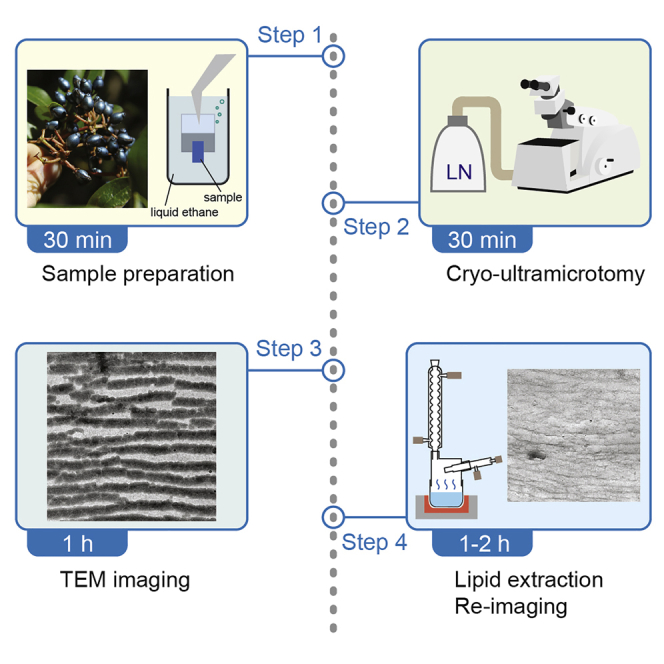

## Before You Begin

Our goal was to characterize the chemical composition of the cell wall of the *V. tinus* fruit, and to develop a procedure that could be applied generally to other systems. We wanted to determine the presence and the spatial organization of lipids in the cell wall at the nanoscale. To do so we developed a technique that enabled us to perform a lipid extraction of the material deposited in grid and visualize the same region of the cell wall before and after the extraction. Specifically, we combine cryo-ultramicrotomy on fresh fruit tissue, low-dose TEM imaging, and a custom-built reflux extractor in order to characterize lipidic nanostructures. The developed methodology avoids (i) using bulk chloroform liquid to extract the lipids, which generally results in the loss of the embedded sections, which often float away; and similarly, (ii) using vaporous or refluxed chloroform which is not effective at lipid extraction and which cause no visible change in the nanostructure.

This procedure has four major steps. In short, we first cut ultrathin sections of fresh (non-embedded) fruit tissue using a cryo-ultramicrotomy procedure described below. Second, we imaged these fresh ultrathin sections with a transmission electron microscope (TEM) to obtain a “before” image. Third, we exposed that tissue to chloroform in order to extract lipids present in the tissue. Finally, we re-imaged the tissue in order to visualize the loss of lipids due to the chloroform extraction. In the following, we describe in more detail how to set up the cryo-ultramicrotome system and the custom reflux extractor for chloroform exposure.

### Setting up the Cryo-Ultramicrotome

**Timing: 1 h**

***Note:*** Make sure that the microtome room is well ventilated and equipped with an oxygen sensor.

**Figure 1:**
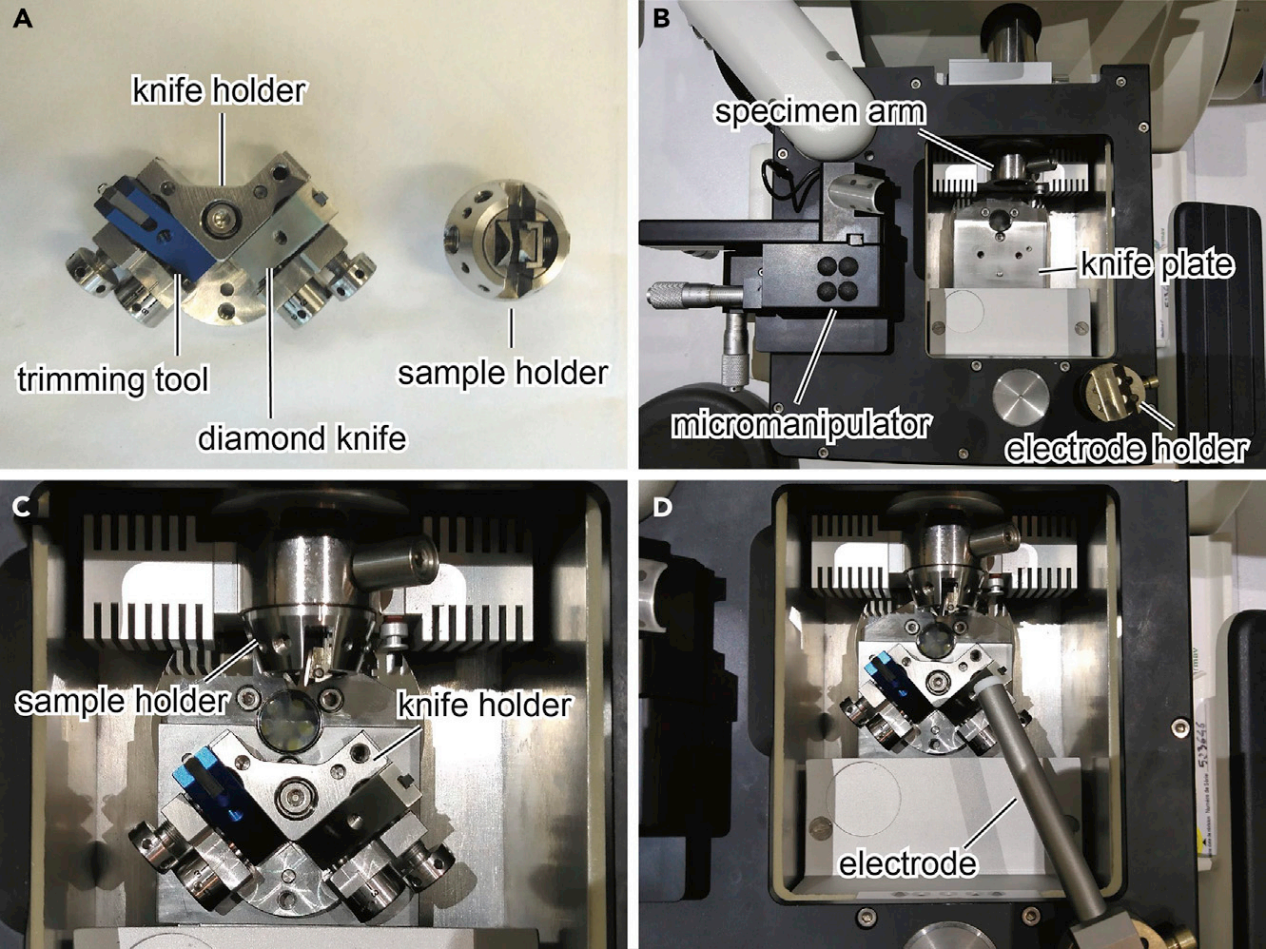
Configuration of the Cryo-Ultramicrotome System (A) The diamond knife and the trimming tool mounted on the rotating knife holder (left) and the sample holder (right). (B) Layout of the cryochamber. (C) The knife and sample holders placed in the cryochamber. (D) The antistatic electrode placed in the cryochamber.
6. Put the rotating knife holder on a knife plate in the cryochamber (Figures 1B and 1C).
7. Insert a specimen holder equipped with a flat clamp in the specimen arm (Figures 1B and 1C).
8. Place an antistatic electrode on the cryochamber and switch on the main power of the antistatic device (Figure 1D).
9. Start cooling of the cryochamber with target temperatures of −90°C for knife and gas temperature and −110°C for specimen temperature. This step takes approximately 30–40 min.
10. Glow discharge a carbon-coated 200-mesh copper grid at 15 mA for 25 s using a glow discharger.

***Note:*** Carbon-coated grids can be purchased (for example, Cat# CF200-Cu from Electron Microscopy Science (EMS)) or prepared using a carbon coater as described elsewhere (Bradley, 1954; Brenner & Horne, 1959). For better tracking of specimen position, use index grids such as Gilder Finder grids (Cat# G200F1-Cu in EMS).

***Note:*** Dry air or nitrogen gas supply is needed for recovery of cryo-sections. If there is no such supply in the microtome room, put a nitrogen gas tank with a regulator next to the microtome.

***Note:*** We did not use a thermal cycler before the cryo-sectioning. Our ambient condition (typically 22°C and 60% RH) did not cause significant water condensation or frost formation.

1. Remove the knife support and armrest from the Ultracut UC6.
2. Mount the FC7 cryochamber to the ultramicrotome Ultracut UC6.
3. Switch on the main power of the ultramicrotome and activate FC7 control option on the control unit.
4. Connect the cryochamber to a liquid nitrogen tank.
5. Mount a cryo trimming tool and a 35° diamond knife on a rotating knife holder (Figure 1A).

### Setting Up Reflux Extractor

**Timing: 30 min**

11. Put the reflux extractor under a fume hood and connect water lines to the condensers.
12. Put about 50 mL of chloroform and a few boiling stones in the solvent container.
13. Place the solvent container at the bottom of the reflux extractor.
14. Start circulating cooling water in the condensers.
15. Place a heat source such as a sand bath under the solvent container to heat it to about 80°C.

## Key Resources Table

**Table:** 

REAGENT or RESOURCE	SOURCE	IDENTIFIER
**Chemicals, Peptides, and Recombinant Proteins**		

Chloroform, CHCl_3_	Sigma-Aldrich	Cat# 288306
Boiling chips granules	Sigma-Aldrich	Cat# 1079130100

**Deposited Data**		

Electron micrograph of untreated cryo-sections	Middleton et al., 2020	N/A
Electron micrograph of CHCl_3_-treated cryo-sections	Middleton et al., 2020	N/A

**Software and Algorithms**		

Fiji	https://imagej.net/Fiji	N/A
SerialEM	https://bio3d.colorado.edu/SerialEM/	N/A

**Other**		

Leica EM UC6	Leica Microsystems	N/A
Leica EM FC7	Leica Microsystems	N/A
35° cryo immuno diamond knife	DiATOME	N/A
Trimtool 45	DiATOME	N/A
Transmission electron microscope JEM 2100Plus	JEOL	N/A
Gatan RIO16 CMOS camera	Gatan	N/A
60-μm In-Gap objective aperture	JEOL	N/A
Reflux extractor	N/A	N/A
Carbon-coated grids	Electron Microscopy Sciences	Cat# CF200-Cu
Gilder Finder grids	Electron Microscopy Sciences	Cat# G200F1-Cu
Graphite adhesive (carbon adhesive paste)	Electron Microscopy Sciences	Cat# 12660
Glow discharger (PELCO easiGlow)	PELCO	Cat# 91000
Stainless steel Dewar	N/A	N/A

**Experimental Models: Organisms/Strains**		

*Viburnum tinus*	N/A	N/A

## Materials and Equipment

### Ultramicrotomy

We used a Leica EM UC6 and a Leica FC7. For sectioning we used a 35° cryo immuno diamond knife and a trimtool 45° (Diatome, USA).

### Imaging

For TEM imaging, we used a transmission electron microscope JEM 2100Plus (JEOL, Japan) operated at 200 kV and equipped with a Gatan RIO16 CMOS camera (Gatan, USA). A 60-μm In-Gap objective aperture was used to enhance the image contrast. The low-dose imaging was performed using Low Dose mode of SerialEM program (Mastronarde, 2003).

### Lipid Extraction

Solubility in organic solvents is a well-established hallmark of lipids (Bligh & Dyer, 1959). We demonstrated the lipidic nature of the globular inclusions of *V. tinus* by chloroform extraction and subsequent TEM imaging that showed the loss of material from the globules after chloroform extraction.

**Figure 2:**
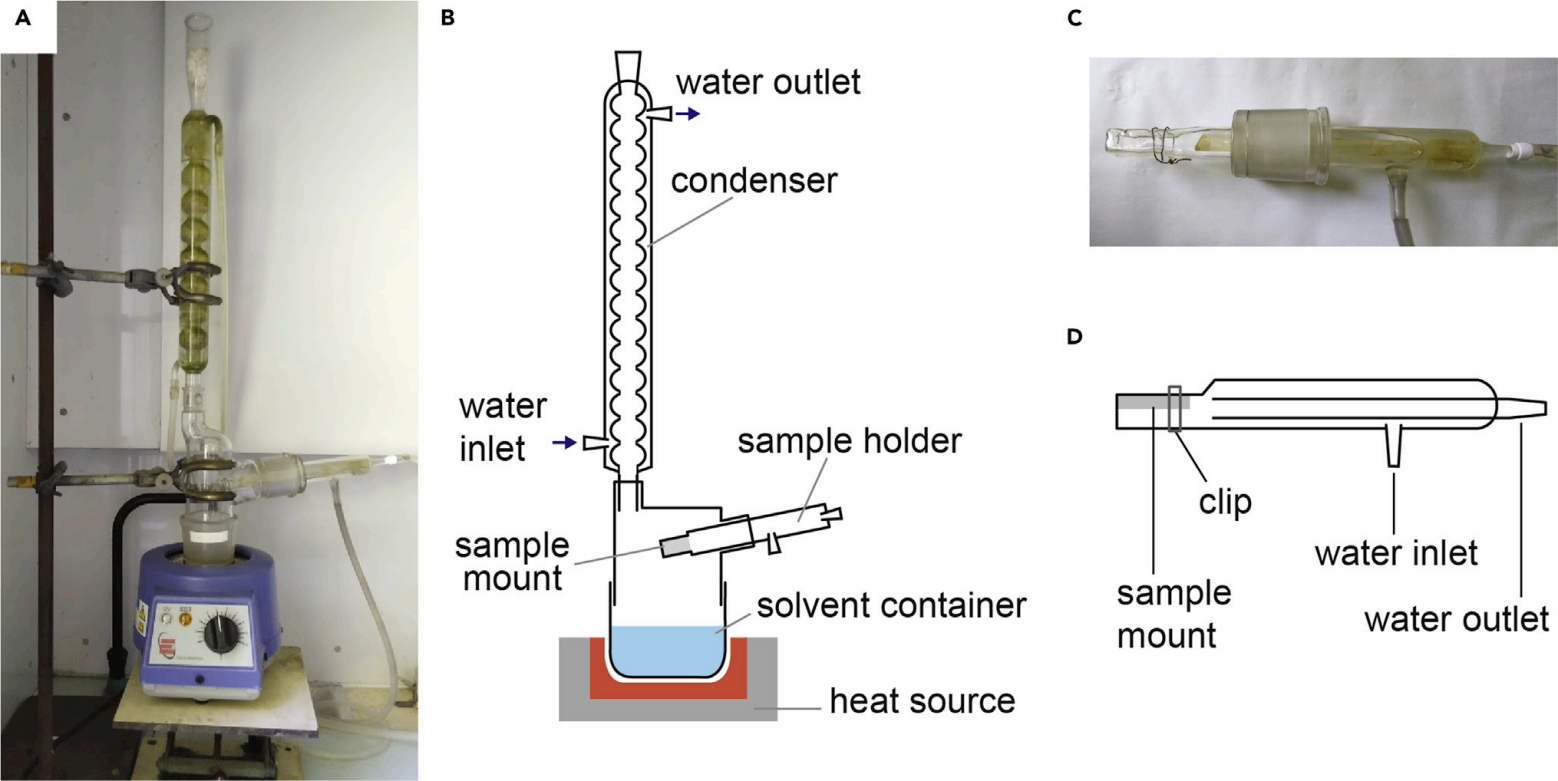
Heat Reflux Extractor with a Water-Cooling Sample Holder (A) Photograph and (B) schematic illustration of the apparatus. (C) Photograph and (D) schematic illustration of the sample holder.

We extracted lipids from cryo-ultrathin sections on EM grids using refluxed chloroform in a reflux extractor shown in Figure 2. This extractor has a side-entry water-cooling sample holder (Figures 2C and 2D). Due to the water cooling, vaporous chloroform condenses to a liquid on the surface of the sample holder, so liquid chloroform gently comes in contact with the sample surface and dissolves and washes away lipids from the sample.

***Alternatives****:* The EM UC6 can be replaced by an EM UC7. The FC7 cryochamber can be replaced by an FC6 cryochamber. A dry cryo diamond knife of any angle could be substituted for the 35˚ knife we used, and the trimtool 45˚ can be replaced by any trimming tool. TEM imaging can be performed with any TEM system with a low-dose imaging function. The customized reflux extractor used in this protocol can be replaced by a two-neck flask with a volume of 200–500 mL, a condenser, and a sample holder with a cooling capability. As can be seen in Figure 2D, the sample holder can be replaced by a double layer glass tube with one closed end.

## Step-by-Step Method Details

We divided these methods into four steps: (1) preparing the sample, (2) performing the cryo-ultramicrotomy, (3) TEM imaging, and (4) lipid extraction. Following this protocol involves TEM imaging both before and after lipid extraction, but the procedure for imaging post-extraction is identical to the procedure pre-extraction.

### Sample Preparation

**Timing: 30 min**

Epicarp of *V. tinus* fruit is cryo-fixed for cryo-ultramicrotomy.

1. Pour liquid nitrogen in a stainless steel Dewar.
2. Put the lower half of a small container in liquid nitrogen and let it cool down to the liquid nitrogen temperature.

**Figure 3:**
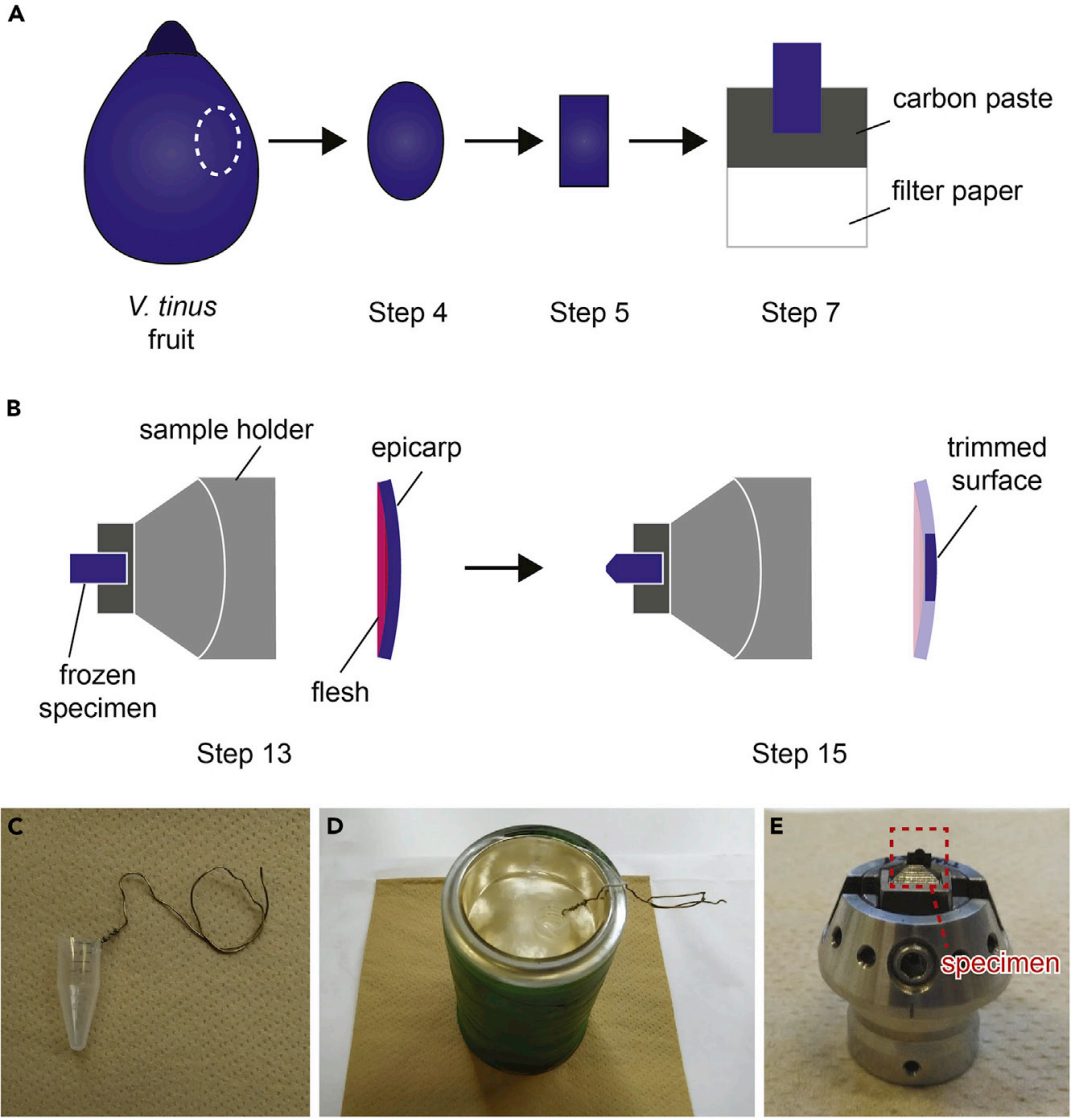
Schematic Illustrations of Sample Preparation (A) Specimen shapes between step 4 and step 7. (B) Side (left) and block face views (right) of the frozen specimen before (step 13) and after (step 15) trimming. (C and D) Freezing apparatus consisting of a centrifuge tube, brass wire, and a Dewar. (E) Specimen placed in a specimen holder. The photo was taken after recovering the specimen to room temperature (ca. 20°C–25°C).

***Note:*** The container may be made of plastic or metal with a volume of about 5 mL. We utilized the lower part of a 15-mL centrifuge tube with a brass wire attached to the edge of the tube as a handle (Figures 3C and 3D).

3. Fill the container with ethane gas and let it liquidize.
4. Peel pericarp of fresh *V. tinus* fruit with a razor blade and blot excess juice with a piece of filter paper.

***Note:*** The blotting time should be minimized, as otherwise it will cause dehydration of the epicarp and alter the ultrastructure.

***Note:*** Thickness of the peeled epicarp is typically 100–200 μm.

***Note:*** While there is little flesh/pulp in *V. tinus*, it is important to remove as much flesh as possible to facilitate the cryo-ultramicrotomy.

5. Cut the peeled epicarp into a strip with dimensions of ca. 3 mm (L) × 1 mm (W), and cut a strip of filter paper with dimensions of ca. 5 mm (L) × 3 mm (W).
6. Spread carbon adhesive paste (graphite adhesive) on half of the filter paper strip.
7. Place the epicarp on the edge of the filter paper strip in such a way that only its lower half is in contact with the half-dried carbon paste on the filter paper as shown in Figure 3A.
8. Let the carbon paste dry and make sure that the pericarp is well adhered to the filter paper strip.
9. Hold the filter paper part of the specimen with tweezers.

***Note:*** Use cross locked tweezers for better handling.

***Note:*** Liquid ethane can solidify in the liquid nitrogen bath. If so, before step 9 melt the solid ethane by touching it with clean room-temperature objects such as tweezers.

10. Quench the specimen in liquid ethane and let it freeze for about 10 s.

***Note:*** This freezing procedure can be performed using an (semi-)automated vitrification machine, such as Leica EMGP or Leica CPC when the specimen size is comparable to the size of an EM grid.

11. Transfer the frozen specimen to liquid nitrogen in a polystyrene container in order to keep it frozen until ultramicrotomy.

### Cryo-Ultramicrotomy

**Timing: 1 h**

Cryo-ultrathin sections of the epicarp are cut and collected on a carbon-coated grid for TEM imaging.

12. Transfer the frozen specimen from the polystyrene container to the cryochamber using tweezers precooled in liquid nitrogen.
13. Fix the filter paper part of the specimen in the flat clamp of the specimen holder (Figures 3C and 3F) and let the specimen temperature equilibrate to the set specimen temperature (−110°C).
14. Rotate the knife holder to put the trimming tool in the cutting position and approach the trimming tool to the tip of the specimen.
15. Trim the specimen using the trimming tool to make a specimen surface with dimensions of ca. 0.1 mm (L) × 0.05 mm (W) (Figure 3B).
16. Rotate the knife holder to set the diamond knife in the cutting position and approach the knife to the specimen surface.
17. Hold the glow-discharged grid with tweezers and insert the tweezers in a micromanipulator of the cryochamber.
18. Using the micrometer gauges of the micromanipulator, place the grid slightly below the diamond knife.
19. Activate the discharge function of the antistatic device.
20. Cut ultrathin sections of thickness of about 90 nm and transfer them to the grid surface using an eyelash. Troubleshooting 1

***Note:*** We used a cutting speed of 0.1 mm/s for ultrathin sectioning.

***Note:*** The ultrathin sections do not form a ribbon in most cases since the shape of the cutting surface is often not ideal due to the tissue shape (as sections are not embedded and thus cannot be shaped into a typical trapezoid), so sections must be individually transferred from the knife edge to the grid surface.

21. Disactivate the discharge function of the antistatic device to ensure adhesion of the sections on the grid surface.
22. Retract the grid from the knife using the micrometer gauges.
23. Remove the grid from the cryochamber and let it warm up to room temperature (ca. 20°C–25°C) under nitrogen gas or dry air flow.

***Note:*** Simple melt-drying is used for the *V. tinus* epicarp since the cell wall ultrastructure is not significantly altered by ice recrystallization due to its robustness and relatively low hydration level. For more hydrated soft tissues, one would need to use gentler recovery methods such as freeze drying to prevent ice artifacts (Geymayer et al., 1978).

### TEM Imaging

**Timing: 1 h**

TEM observation is performed on the cryo-ultrathin sections of the epicarp under low-dose condition.

24. Mount the grid in a standard TEM holder and then insert the holder into the TEM.
25. Adjust the sample position to the eucentric height and perform TEM alignment if necessary.
26. Set up Low Dose mode in SerialEM at a blank (non-specimen) area. The overall radiation dose, a sum of those from Search and Acquisition, should not exceed 4 e-/Å^−1^.

**Figure 4:**
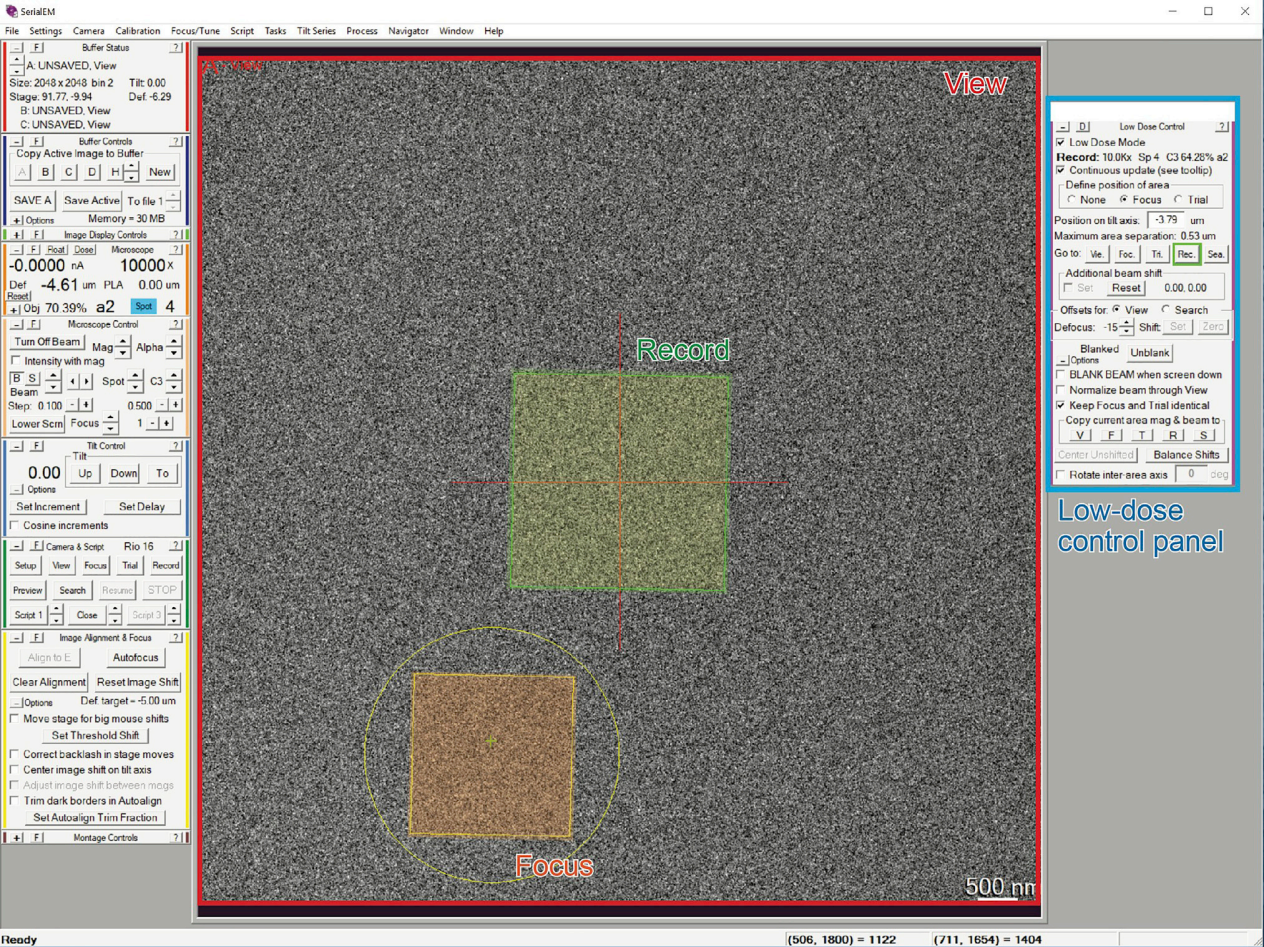
Low-Dose Setting in SerialEM Colored squares indicate three predefined areas in the low-dose mode.

***Note:*** For better tracking of the sections after the lipid extraction step it is recommended to take a low magnification image of the sections (×100–600). Because the same area of the sample should be re-imaged following lipid extraction with chloroform, the sample will likely be rotated in the grid holder. A low magnification image facilitates finding the same region of the sample for re-imaging.

***Note:*** The detailed procedure for setting up of Low Dose mode can be found in the online help of SerialEM (https://bio3d.colorado.edu/SerialEM/hlp/html/about_low_dose.htm). Briefly, the Low Dose mode consists of three “areas”: Record, View, and Focus, as shown in Figure 4. The Record area is the area where a micrograph is recorded. The View area is at lower magnification and shares the center of the Record area. The Focus area is used to check and modify the defocus value without exposing the Record area, so it is displaced from the Record area along the tilt axis. In this protocol, the magnification was set at ×6,000–10,000 for Record and Focus, and ×2,000–4,000 for View. The target defocus value was set at −5–10 μm.

27. Acquire images of the sections using the Low Dose mode.

**CRITICAL:** The specimen is an unfixed and unstained biological tissue, so extensive electron radiation causes severe radiation damage which alters the chemical composition of the components, and hence their solubility to solvents. It is thus necessary to perform TEM imaging in the low dose condition to minimize the radiation damage.

**Figure 5:**
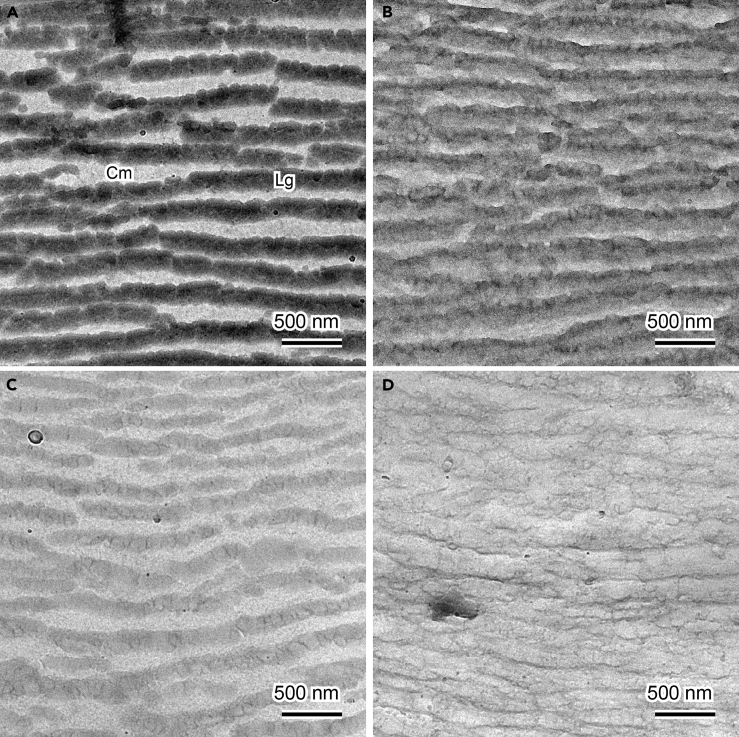
Image Contrast of Lipid Globules in the Epicarpal Cell Wall of *V. tinus* Before and After Chloroform Extraction (A) Untreated globules showing strong dark image contrast. Cm, cell wall matrix; Lg, lipid globules. (B) Globules after insufficient chloroform extraction with slightly reduced image contrast. The contrast is heterogeneous in a single globule due to partial dissolution of lipids. (C and D) Globules after sufficient chloroform extraction. The image contrast of the globules is homogeneously and significantly reduced (C) or almost absent (D). Scale bars, 500 nm.

***Note:*** In the case of *V. tinus*, the unstained lipid globules show dark image contrast due to their crystallinity and likely higher density compared to that of the surrounding cell wall matrix (Figure 5A).

28. Remove the grid from the TEM.

### Lipid Extraction and Re-imaging

**Timing: 1–2 h**

Lipids in the epicarp are extracted using the reflux extractor and the treated section is observed to localize the lipids.

29. Put the grid on a filter paper strip.
30. Put the strip on the sample holder of the reflux extractor.
31. Insert the holder in the extractor and tilt the holder by about 20°, so condensed chloroform will flow down from the holder surface.
32. Treat the grid with refluxed chloroform for 10–20 min.

**CRITICAL:** Make sure that condensed chloroform flows down from the sample holder. The movement of liquid chloroform around the specimen is essential for effective extraction of lipids. Troubleshooting 2

33. Remove the grid from the extractor and let it dry in air.
34. Insert the treated grid into the TEM.
35. Observe the previously observed area of the specimen under the same low dose condition.
36. Compare the image contrast of the same view field before and after the chloroform extraction.

***Note:*** After the chloroform extraction the loss of material of the lipid globules should be observed as their reduced image contrast as shown in Figure 5.

## Expected Outcomes

At the end of process, the localization of lipids in the epicarp should be revealed at the TEM resolution, as demonstrated in Figure 3 of Middleton et al. (2020). The gentle chloroform extraction procedure preserves the ultrastructure, which allows a direct comparison of EM images of identical sections before and after the chloroform treatment.

***Note:*** We did not experience any severe ice artifacts likely due to the robustness of the cell wall architecture.

## Limitations

In this protocol, lipid localization is determined based on the change in TEM image contrast. Thus, a lipidic structure of interest must produce enough image contrast without contrasting agent. Additionally, structures of studied tissues must be relatively stable because they are not chemically fixed and undergo several temperature changes. This should be the case for most plant tissues due to the presence of cell wall architecture. For this reason, this protocol should be also applicable to other biological specimens with robust cell architectures, such as fungi and yeasts. The solubility of lipids may differ due to various factors, such as their molecular weight, crystallinity, and the thickness of the ultrathin sections.

## Troubleshooting

### Problem 1

Difficulty achieving sufficiently ultrathin cryo-sections of the fruit epicarp (step 20).

### Potential Solution 1

Cryo-ultrathin sectioning of the epicarp can be difficult due to various factors, such as the water content of the specimen, the amount of frozen flesh and excess juice, and the size of the cutting surface. Trimming out most of the frozen flesh and juice improves the homogeneity of mechanical properties of the specimen. Setting T_specimen_ down to around −130°C can further stiffen the specimen and improve its cuttability.

### Problem 2

Lipids do not extract in 20 min of chloroform exposure (step 32).

### Potential Solution 2

We observed variations in the treatment time required for the effective lipid extraction from 10 min to 1 h. This difference might originate from variation in the section thickness or accessibility of lipid molecules, such as due to the presence of the membrane structure around the lipid globules. Insufficient extraction results in slightly reduced image contrast of lipidic structures as shown in Figure 5B. In such a case, one can repeat the procedure from step 29 to 35 until significant contrast reduction is observed (Figures 5C and 5D). Note that the TEM imaging has to be performed under the low dose condition to avoid radiation-induced chemical modifications of lipids as aforementioned.

## Resource Availability

### Lead Contact

Further information and requests for resources and reagents should be directed to and will be fulfilled by the Lead Contact, Dr Yu Ogawa (yu.ogawa@cermav.cnrs.fr).

### Materials Availability

This protocol did not generate any new material.

### Data and code Availability

This protocol did not generate any new data or code.
